# Structural Insights into Phosphorylation-Mediated Polymerase Function Loss for DNA Polymerase *β* Bound to Gapped DNA

**DOI:** 10.3390/ijms24108988

**Published:** 2023-05-19

**Authors:** Amit Srivastava, Haitham Idriss, Dirar Homouz

**Affiliations:** 1 Department of Physics, Khalifa University of Science and Technology, Abu Dhabi 127788, United Arab Emirates; amit.srivastava@ku.ac.ae; 2 School of Public Health, Imperial College of Science, Technology and Medicine, London SW7 2AZ, UK; haitham.idriss19@imperial.ac.uk; 3 Palestinian Neuroscience Initiative, Al-Quds University, Jerusalem 51000, Palestine; 4 Faculty of Health Sciences, Global University, Beirut 15-5085, Lebanon

**Keywords:** DNA polymerase β, post-translational modifications, MD simulations, principal component analysis

## Abstract

DNA polymerase β is a member of the X-family of DNA polymerases, playing a critical role in the base excision repair (BER) pathway in mammalian cells by implementing the nucleotide gap-filling step. In vitro phosphorylation of DNA polymerase β with PKC on S44 causes loss in the enzyme’s DNA polymerase activity but not single-strand DNA binding. Although these studies have shown that single-stranded DNA binding is not affected by phosphorylation, the structural basis behind the mechanism underlying phosphorylation-induced activity loss remains poorly understood. Previous modeling studies suggested phosphorylation of S44 was sufficient to induce structural changes that impact the enzyme’s polymerase function. However, the S44 phosphorylated-enzyme/DNA complex has not been modeled so far. To address this knowledge gap, we conducted atomistic molecular dynamics simulations of pol β complexed with gapped DNA. Our simulations, which used explicit solvent and lasted for microseconds, revealed that phosphorylation at the S44 site, in the presence of Mg ions, induced significant conformational changes in the enzyme. Specifically, these changes led to the transformation of the enzyme from a closed to an open structure. Additionally, our simulations identified phosphorylation-induced allosteric coupling between the inter-domain region, suggesting the existence of a putative allosteric site. Taken together, our results provide a mechanistic understanding of the conformational transition observed due to phosphorylation in DNA polymerase β interactions with gapped DNA. Our simulations shed light on the mechanisms of phosphorylation-induced activity loss in DNA polymerase β and reveal potential targets for the development of novel therapeutics aimed at mitigating the effects of this post-translational modification.

## 1. Introduction

More than 10,000 instances of spontaneous DNA damage occur daily in every human cell [1]. The impact of unrepaired DNA damage on cell function can have far-reaching consequences, including the development of cancer or neurodegenerative disease [2,3,4]. The precise synthesis and upkeep of DNA require an intricate network of proteins, with DNA polymerase enzymes playing a particularly vital role. DNA polymerases catalyze template-dependent DNA synthesis during cell repair and replication. These enzymes are responsible for selectively binding and incorporating nucleotides from a pool of chemically and structurally similar molecules, ensuring that the correct nucleotide is paired with the corresponding template base. Moreover, replicative DNA polymerases can synthesize DNA at rates ranging from tens to hundreds of nucleotide additions per second, with a relatively low error rate of misincorporation occurring only once every ten to hundred of thousands of nucleotides added [5]. The selectivity observed in DNA polymerases indicates that the conformational dynamics of these enzymes play a crucial role in substrate binding and catalysis. However, many aspects of the structure, function, and dynamics of DNA polymerases remain incompletely understood [6].

DNA polymerases are classified into different families, including A, B, C, D, X, and Y. Pol β, a member of the X family, is the smallest nuclear DNA polymerase and is responsible for gap filling, which is the penultimate step in critical cellular processes, within mammalian cells [7]. Pol β is a structurally and functionally attractive model for investigating the mechanisms involved in efficient DNA synthesis by polymerases. Several high-resolution crystal structures have been reported for DNA pol β in both binary and ternary complexes [8,9,10]. DNA pol β consists of 335 residues and comprises two domains: an 8 kD N-terminal domain that displays deoxyribose phosphate lyase activity and a 31 kD C-terminal domain that possesses nucleotidyl transfer activity. Similar to other DNA polymerases, the polymerase domain of pol β has a modular organization consisting of three subdomains that serve different functions. These subdomains include the DNA binding D-subdomain (residues 90–150), the catalytic C-subdomain (residues 151–260), and the nascent base-pair-binding N-subdomain (residues 261–335), which correspond to the thumb, palm, and fingers subdomains, respectively. The C-subdomain coordinates two divalent metal cations (Mg${}^{2+}$) that facilitate DNA synthesis. The other two subdomains, D and N, are spatially situated on opposite sides of the C-subdomain. Although the mechanism of substrate binding and product release by DNA polymerases has been well-studied, the conformational changes involved in these processes have not been fully elucidated. Methods based on molecular dynamics (MD) simulations have been employed to answer important questions regarding DNA pol β, such as structural changes, mutagenic lesions, and fidelity maintenance [11,12,13]. Although molecular dynamics (MD) simulations have shed light on subdomain motions, the mechanism underlying the transition of the DNA polymerase complex from the closed to the open state remains unclear [14,15,16].

In the closed ternary complex of DNA pol β, one Mg${}^{2+}$ ion coordinates with incoming deoxy-nucleotide triphosphate (dNTP), and the other Mg${}^{2+}$ (the catalytic ion) is positioned between the incoming dNTP and 3${}^{\prime }$-terminus of primase. Both magnesium ions are anchored to the active sites by coordinating highly conserved acidic residues (i.e., ASP${}^{190}$, ASP${}^{192}$, and ASP${}^{256}$) [17,18]. The mechanism involving two magnesium ions is universal for nucleotide addition and is observed in both DNA and RNA polymerases.

Molecular dynamics (MD) simulation studies on various proteins have shown that post-translational modifications, particularly phosphorylation, can induce conformational changes in the protein [19,20]. Experimental studies have reported that phosphorylated DNA pol β retains its ability to bind ssDNA but loses its polymerase activity in vitro after undergoing serine phosphorylation (S44 and S55) with protein kinase C (PKC). It is widely accepted that the presence of Mg ions is required for the accurate positioning of incoming dNTPs with template nucleotides in the gap that needs to be filled [21,22]. Recently, we investigated the effect of Mg ions and examined the impact of phosphorylation on the stability of the enzyme when phosphate atom was added to S44 [23]. Our investigation revealed that phosphorylation at S44 induces conformational changes in DNA pol β, affecting the movement of various subdomains, including the lyase and base-pair binding subdomains. Furthermore, we observed the formation of stable salt bridges upon phosphorylation in the presence of Mg ions. To better understand the effect of phosphorylation on the DNA polymerase complex, it is essential to simulate the entire complex, which includes both the DNA and polymerase protein. In our previous study, we only considered the DNA polymerase protein and Mg ions to simplify the system. Therefore, we aim to expand on our previous studies and investigate how the phosphorylation of DNA pol β affects its ternary complex. We introduced a dianionic phosphoserine (SP2) patch to model the phosphorylated serine. Exploring the interaction between the newly added phosphate and the backbone phosphates of DNA would be a fascinating avenue for this study.

Previous studies using molecular dynamics (MD) simulations have extensively investigated the fidelity of DNA polymerase and conformational changes observed in kinetic experimental studies [24,25]. However, limited attention has been paid to the impact of mutations on subdomain motion in the DNA pol β complex and the effect of mutations [26] on the protein’s free energy landscape (FEL). Furthermore, the impact of post-translational modifications, particularly phosphorylation, on the activity and conformational changes of the DNA pol β ternary complex has yet to be investigated. In this study, we conducted thorough MD simulations, lasting up to microsecond time scales, to investigate the conformational changes of both wild-type and phosphorylated DNA pol β complexes in the presence of divalent Mg and monovalent Na ions. The phosphorylated serine residue was modeled using an SP2 patch. Our primary objective is to determine the impact of phosphorylation on the structural dynamics of the DNA pol β complex and, in particular, to examine how the conformational changes of the complex affect the motion of its subdomains in the presence of both types of ions. Additionally, we investigated the interplay between the different subdomains of the enzyme induced by phosphorylation. Our findings provide novel insights into the mechanism underlying activity loss and conformational transitions in the DNA pol β complex caused by phosphorylation.

## 2. Results and Discussion

**Figure 1:**
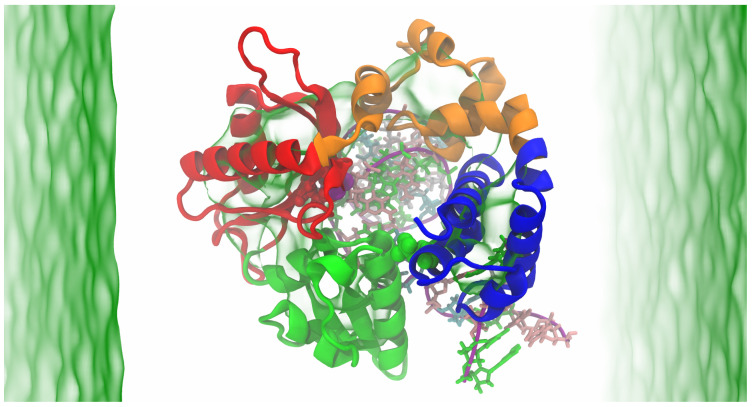
Schematic representation of DNA polymerase β complex. The protein is in the closed conformation. The protein structure includes the lyase domain (residues 10–87) (blue color) and three subdomains: DNA binding (D, residues 90–150) (orange color), catalytic (C, residues 151–260) (red color), and nascent base-pair-binding (N, residues 261–335) (green color). These correspond to the thumb, palm, and fingers subdomains, respectively, of DNA polymerases. The DNA is shown in purple.

The DNA polymerase β complex undergoes several conformational changes during nucleotide incorporation along its reaction pathway, with crystal structures of most of the intermediate states resolved experimentally. The unliganded pol β assumes an extended structure (PDBID:1BPD [27]), which transitions to an open structure upon binding DNA (1BPX [28], 3ISB [29]). Further conformational changes occur upon binding dNTP, leading to the closed state, where the N-terminal subdomain rotates (PDBID:2FMS) [30]. A schematic diagram of the simulated system is depicted in Figure 1. This study investigates the impact of phosphorylation on the structural and dynamic properties of the DNA pol β complex, using molecular dynamics (MD) simulations in the presence of Mg ions that assist the DNA pol β to transition into a closed state prior to the chemical reaction. The number in the parentheses is the standard deviation of that reported parameter metric.

### 2.1. Structural Stability and Flexibility Analysis

**Figure 2:**
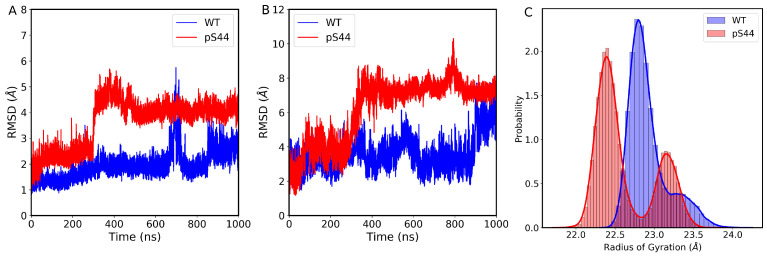
(**A**) The evolution of root mean square deviation (RMSD) of $C_{\alpha }$ atoms with respect to the initial structure of WT (blue color) and pS44 (red color). (**B**) Time evolution of RMSD of the backbone atoms of DNA fragment with respect to the initial structure WT (blue) and pS44 (red). (**C**) Probability distribution of radius of gyration $R_{g}$ of DNA polymerase β complex: WT (blue) and pS44 (red).

To assess the structural changes, overall stability, and convergence of our simulated system, we computed the root mean square deviations (RMSDs) of $C_{\alpha }$ atoms for protein and backbone phosphate for DNA from the initial structure. Figure 2A shows the time evolution of RMSD of $C_{\alpha }$ atoms of protein with respect to the initial structure of DNA polymerase both for WT and pS44. Based on Figure 2A, it can be observed that the RMSDs of both the WT and pS44 systems show initial fluctuations but eventually stabilize after 300 ns, indicating convergence of the simulation trajectories. Notably, there is a sudden increase in the RMSD value of the pS44 system at 310 ns, where it jumps up to 5.8 Å before reaching a plateau after 500 ns. In contrast, the WT system reaches equilibrium after 250 ns and remains stable until 650 ns. At 680 ns, there is a sudden jump in RMSD with a value of 5.6 Å, which gradually returns to the initial state at around 800 ns and remains stable for the rest of the simulation time. The figure clearly illustrates that phosphorylation makes the system more flexible. The mean RMSD values for pS44 and WT were calculated to be 3.62 (0.64) Åand 1.93 (0.53) Å, respectively. Figure 2B shows the time evolution of RMSD of backbone atoms of DNA fragments with respect to the initial structure of DNA polymerase both for WT and pS44. Similar to the protein RMSDs, the DNA backbone also exhibits a similar pattern. The average RMSD value of the DNA fragment for the WT system is 2.86 (0.95) Å, while for the pS44 system, the average RMSD value is 6.14 (1.4) Å. The higher RMSD value observed in the DNA fragment of pS44 compared to protein is due to the interaction between the P atom of the phosphorylated S44 residue and the phosphate atom of the DNA backbone. In contrast to our previous work [23], where DNA was absent, the presence of DNA in the current system results in higher stability. However, the RMSD values decrease in both cases for the protein.

The phosphorylation effect on the structural compactness of the DNA pol β complex can be examined by computing the radius of gyration ($R_{g}$). Figure 2C shows the frequency distribution of $R_{g}$ of WT and pS44. The distribution of $R_{g}$ appears to have two distinct peaks for both the WT and pS44 cases. In the case of WT, the two peaks are somewhat merged, with values of approximately 22.79 and 23.22 Å, while for pS44, the two peaks are more distinguishable, with values of approximately 22.19 and 23.19 Å. The presence of these peaks suggests the occurrence of conformational transitions. In order to further investigate these transitions, we calculated the free energy profiles for both systems.

**Figure 3:**
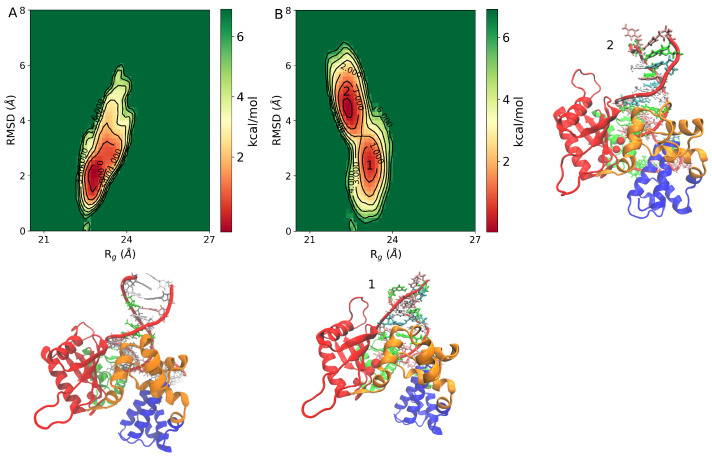
Free energy landscape of DNA polymerase β as a function of $R_{g}$ (Å) and RMSD (Å) (**A**) WT, and (**B**) pS44. The representative structure corresponds to minimum energy basins are also shown in the figure. For pS44, the minimum free energy basins are highlighted using the numbers.

The conformational changes in DNA pol β complex are explored by the FEL. The FEL is projected along the two reaction coordinates i.e., RMSD ($C_{\alpha }$ and backbone phosphate atom for DNA) and $R_{g}$ of DNA pol β complex, which is shown in Figure 3. The FEL provides a representation of the conformational space explored by the DNA pol β complex. The FEL plot indicates that in the pS44 system, there are two distinct minimum free energy basins which are characterized by RMSD∼2.26 Å/$R_{g}$∼22.8 Å and RMSD∼4.2 Å/$R_{g}$∼21.98 Å, respectively, while for the WT system, there is only a single minimum free energy state explored, characterized by RMSD∼2.11 Å/$R_{g}$∼22.5 Å. This indicates that the pS44 system exhibits more conformational deviations compared to the WT system. The snapshots shown in Figure 3A (WT) and Figure 3B (pS44) correspond to the free energy basins for both cases. The figures demonstrate that the system, due to phosphorylation, visits the conformational states that correspond to the peaks observed in Figure 2. The two energy basins correspond to different orientations of the DNA binding subdomain, as shown in the orange color in the snapshots. In the first basin, the subdomain is in the closed state, while in the second basin, it twists towards the catalytic subdomain. These observations suggest that phosphorylation affects the function and structure of the DNA binding subdomain and supports the opening and closing mechanisms described in previous research [21]. Despite these conformational changes, the value of $R_{g}$ is not significantly affected by phosphorylation, as the changes involve movements of the DNA binding subdomain towards other subdomains.

**Figure 4:**
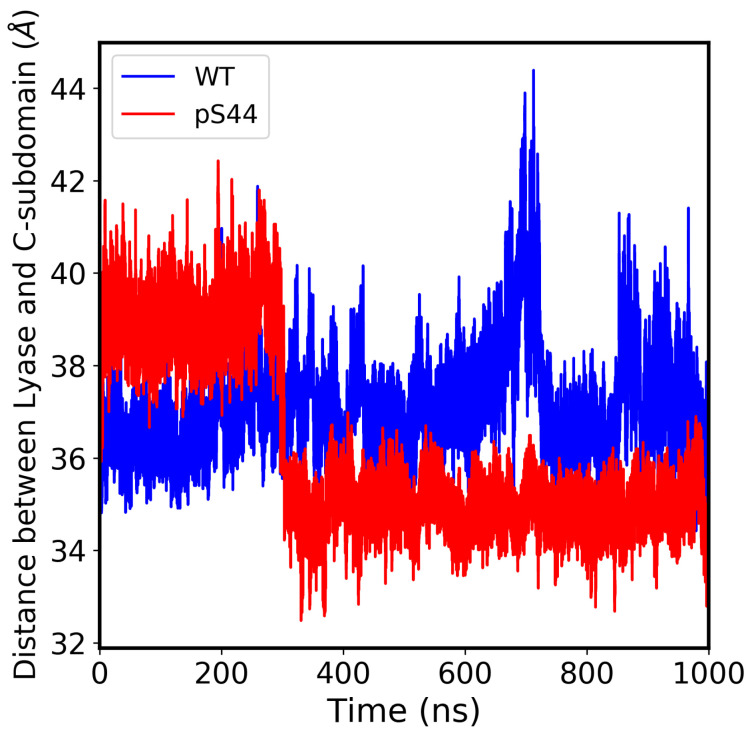
Distance between the lyase domain and catalytic subdomain vs. simulation time.

To further illustrate this, Figure 4 shows that pS44 pol β’s lyase domain lies closer to the C-subdomain. The lyase domain has a helix–hairpin–helix (HhH) motif (residues 55–79) that interacts with the DNA backbone in a sequence-independent manner. This HhH, along with another HhH from the D-subdomain, also interacts with the DNA backbone and serves to stabilize the bent gapped DNA molecule in position on the enzyme prior to the gap filling. This also positions the nascent base pair between the primer terminus base pair and α-helix N of the N-subdomain [22]. Phosphorylation of S44 likely disrupts pol β’s lyase HhH–DNA backbone interaction because it causes a dislocation of the lyase domain towards the C-subdomain. Prior to catalysis, at least two active site positively charged arginine residues (R149, and R183) interact with the negatively charged phosphates of the incoming nucleotide [31]. We hypothesize that the addition of phosphates on S44 alters this interaction disrupting the proper alignment of the incoming dNTP with gapped DNA.

Moreover, Figure S1 (in SI) shows that upon phosphorylation the pS44 becomes closer to R149 but not R183 (as a consequence of the lyase domain moving closer toward the C-subdomain). R149 and R183 are part of a positively charged pocket that interacts with the incoming dNTP substrate. The presence of these two highly conserved arginine residues helps to stabilize the binding of the negatively charged phosphate groups of the incoming dNTP, ensuring proper positioning of the substrate for catalysis. We hypothesize that the negatively charged phosphate of pS44 engages the positive charge on the side chains of arginine 149 making it unavailable to engage with the dNTP negatively charged phosphates. Consequently, this destabilizes the incoming dNTP leading to the failure of polymerase enzyme catalysis.

**Figure 5:**
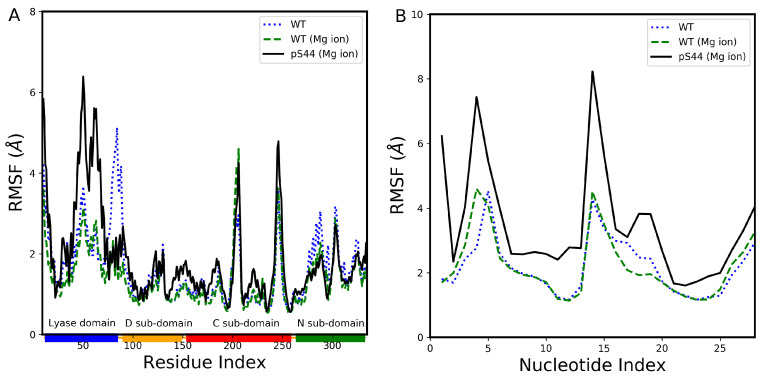
The root mean squared fluctuations (RMSF). (**A**) WT (dotted line), WT with Mg ion (dashed line), and pS44 (solid line). The rectangles shown in the horizontal panel reflect the DNA polymerase major subdomains and are colored as follows: lyase domain (blue) and three subdomains, D (orange), C (red), and N (green). (**B**) RMSF of phosphate atom of DNA backbone: WT (dotted line), WT with Mg ion (dashed line), and pS44 (solid line).

The phosphorylation at S44 residue site induces large structural changes in the closed structure of pol β complex. To identify the subdomains affected by the phosphorylation, we have calculated the root mean square fluctuation (RMSF) for $C_{\alpha }$ atoms of protein residues and backbone phosphate atoms of DNA. The RMSF value shows the average fluctuation of each considered atom over the total time of the simulation. The RMSF values of the protein for pS44 (solid line), WT with Mg ion (red dashed line), and WT (blue dotted line) are displayed in Figure 5A. It can be observed from the figure that the Mg ion, located near the active sites, stabilized the protein by reducing the RMSF fluctuations, except for the linker region between domains. Phosphorylation caused significant structural alterations in the lyase domain of the pol β complex. It led to an increase in fluctuations in the helix region of the DNA binding domain and the catalytic subdomain, while the N-subdomain remained unaffected. This is contrary to our previous study [23] where DNA was absent. We also investigated the impact of phosphorylation on the DNA fragment. Figure 5B displays the RMSF values of the DNA fragment for WT, WT with Mg ion, and pS44. It is evident from the figure that the inclusion of Mg ions does not significantly alter the RMSF value of the DNA fragment, but there is a significant increase in fluctuations after phosphorylation. This increase in fluctuations could be due to the phosphate atom of the phosphorylated site interacting with the DNA backbone atoms, resulting in greater fluctuations through the repulsion of the negative charge of the DNA and S44 phosphates.

The crystal structure of the DNA polymerase complex revealed that the enzyme adopts a closed state due to the presence of a hydrogen bond between S44 and E335 residues, which is absent in an open state. This suggests that this interaction is responsible for stabilizing the enzyme in the closed state. To investigate the effect of S44 phosphorylation on this bond, we analyzed the H-bond occupancy during the MD simulation. The H-bond occupancy is defined as the fraction of time in which the S44 residue forms the H-bond with the E335 residue. The Gromacs hbond tool was used to calculate the presence of the H-bond using a threshold distance of 3.5 Å and a cutoff angle of $30^{\circ }$. According to the results, the H-bond was present in the WT system for 80% of the simulation time (Figure S2 in the SI), while in the phosphorylated S44 system, the H-bond was disrupted, and its occupancy remained at 0.0 throughout the simulation. The average donor–acceptor distance between S44 and E335 for all simulated systems is presented in Table S1 in the SI. The disruption of the H-bond might be attributed to the negative charge of the phosphate group.

We further analyzed the impact of S44 phosphorylation on polar interactions by calculating the H-bonds formed between pS44 and the different domains of the DNA pol β complex. Our results showed that the number of H-bonds varied throughout the simulation. Notably, the lyase domain formed an average of 5.31 (0.8) H-bonds with pS44, while the D subdomain formed 1.43 (0.9) H-bonds, and the N-subdomain formed 0.0038 (0.06) H-bonds. Since pS44 is located in the Lyase domain, it forms more H-bonds with neighboring residues, leading to increased intra-subdomain stability compared to the WT. There were no H-bonds formed between the catalytic subdomain and DNA fragment with the pS44 residue, whereas the average number of H-bonds between the Lyase and S44 residue for WT was 1.72 (0.6). To investigate whether phosphorylated residues affect the DNA fragment, we calculated the distance between pS44 and DNA. The average distance between the pS44 residue and DNA is 24.2 (1.1) Å, whereas for the WT system, the average distance is 26.6 (1.2) Å. This indicates that phosphorylation results in the DNA moving closer to the phosphorylation site. Figure S3 (in SI) depicts the distance between S44 and DNA for both WT and pS44. Our previous study elaborated on the inter-subdomain interactions through H-bonds. Figure S4 (in SI) illustrates how DNA influences the subdomain interaction in the DNA pol β complex. An additional question arises regarding whether phosphorylation affects DNA-protein interactions. To address this, we computed the H-bonds formed between DNA and various subdomains. For the pS44 system, the average number of H-bonds between DNA and D-subdomain is 4.8 (1.2); DNA and C-subdomain is 4.6 (1.1); DNA and N-subdomain is 2.7 (0.6). In contrast, for WT, the average number of H-bonds between DNA and D-subdomain is 2.7 (0.9); DNA and C-subdomain is 4.6 (1.1); DNA and N-subdomain is 2.7 (0.6). These findings suggest that phosphorylation results in the DNA moving closer to the DNA-binding subdomain due to newly formed H-bonds that override any repulsion between the negatively charged pS44 and DNA phosphates. This agrees with experimental findings that DNA binding phosphorylated enzyme was not compromised. The time evolution of hydrogen bond formation between the DNA and different subdomains for both cases is shown in Figure S5 (in SI), highlighting the role of hydrogen bonds in the structural transition observed in the pS44 system’s FEL.

### 2.2. Newly Formed Salt Bridges Due to the Phosphorylation

**Figure 6:**
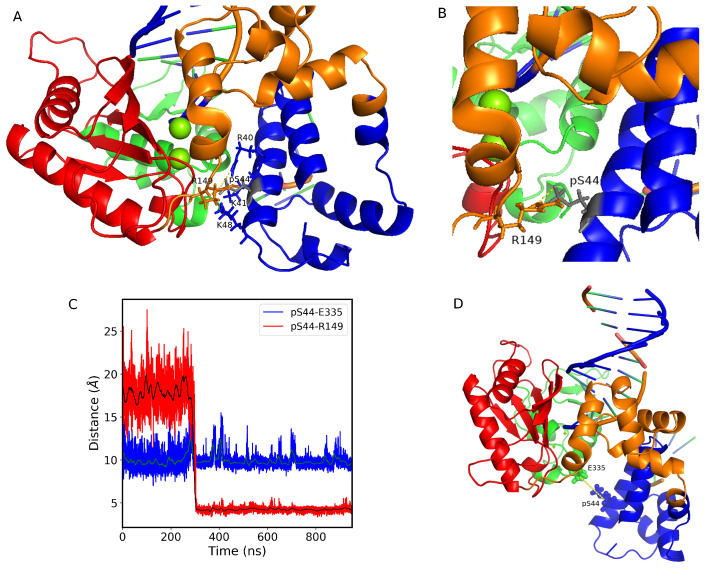
(**A**) Cartoon representation of salt bridge form with residues R40, K41, K48, and R149. The residues pS44, R40, K41, K48, and R149 are shown in stick representation. (**B**) Salt bridge formed with R149 is shown by zooming the structure. (**C**) The pS44-E335 (blue) and pS44-R149 (red) residue distances vs. simulation time. The green and black solid lines show the moving average of data with a bin size of 500. (**D**) Cartoon representation of pS44-2FMS structure. The pS44 and E335 residues are shown in stick representation. DNA polymerase β major subdomains are colored as mentioned in Figure 1.

The lack of a hydrogen bond between S44 and E335 caused by phosphorylation leads to the pol β complex being in an open state. Consequently, the phosphorylated S44 residue is expected to form new salt bridges with neighboring residues and different subdomains. We conducted a salt bridge analysis in the presence and absence of Mg ions. In the absence of Mg ions, the phosphorylated S44 forms five salt bridges (R40, K41, K48, K280, and H285). However, in the presence of Mg ions, only four salt bridges form with residues (R40, K41, K48, and R149), as shown in Figure 6A. The first three residues (R40, K41, and K48) are located in the lyase domain and are unlikely to cause significant structural changes in the pol β /DNA complex upon salt bridge formation. The addition of Mg ions disrupts two new salt bridges (K280 and H285, located in the N-terminal subdomain) formed due to phosphorylation and forms a new salt bridge with R149, which is located in the coil region that connects the D subdomain to the C subdomain (Figure 6B). To investigate the effect of Mg ions on salt bridge formation, we calculated the average O-N distances between the phosphorylated S44 residue and the R40, K41, and K48 residues in the presence and absence of Mg ions. We observed that adding Mg ions decreases the distance between these residues. In the pS44 system with Mg ions, the average distance between S44 and R40 is 4.56 Å, between S44 and K41 is 5.54 Å, and between S44 and K48 is 3.46 Å, while in the absence of Mg ions, the distances between pS44 and these residues are 4.36 Å, 7.76 Å, and 3.62 Å, respectively. These results indicate that Mg ions induce the movement of DNA towards the DNA binding domain, leading to a more compact lyase domain.

In a previous study, it was noted that in the absence of DNA, the phosphorylated S44 residue formed six salt bridges with various residues, including R40, K41, K48, R149, K280, and R299. However, in the presence of DNA, the salt bridges with K280 and R299, which are located in the N-subdomain, were absent. This is because the DNA molecule moves closer to the DNA binding domain, obstructing communication between the lyase domain and N-subdomain. The O-N distances between pS44 and residues K280 and R299 are illustrated in Figure S6, along with a comparison to the distances observed in the absence of DNA.

Our findings reveal that phosphorylation of S44 triggers the creation of a new salt bridge involving residue R149, which is located in the coil region that connects the D-subdomain to the C-subdomain. This newly formed salt bridge is solely observed in the presence of Mg ions. Moreover, our analysis demonstrates a significantly low correlation between the O-N distances of pS44–R149 and pS44–E335 (as shown in Figure 6C,D), which is likely attributed to the structural modification induced by the formation of the new salt bridge. These two competing interactions appear to be the primary drivers behind the structural alterations seen in the DNA pol β complex. Taken together, our results suggest that the presence of Mg ions plays a crucial role in facilitating the formation of these salt bridges.

### 2.3. Phosphorylated S44 Enhances the Correlated Motions in Subdomains of DNA Polymerase β Complex

**Figure 7:**
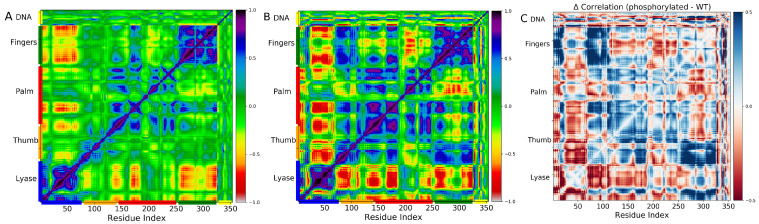
Dynamic cross-correlation matrices of the fluctuations of the $C_{\alpha }$ atoms and phosphate atoms of DNA pol β complex around their equilibrium positions for (**A**) WT and (**B**) pS44 are shown, respectively. The correlated motions of the residues are color-coded (see the scale on right, highly correlated = 1, not correlated = 0, and anti-correlated = −1). (**C**) The difference in correlation matrix element for pS44 with respect to WT. The phosphorylation enhances the correlation between the thumb and finger subdomains.

We utilized a method outlined in the method section to explore the impact of phosphorylation on the subdomain movements of the pol β complex by calculating dynamic cross-correlation maps. In this analysis, we considered protein $C_{\alpha }$ atoms and phosphate atoms of the DNA backbone. The resulting cross-correlation maps for both WT (Figure 7A) and pS44 (Figure 7B) are presented. Figure 7C illustrates the variation in the correlation matrix element for pS44 compared to WT. The figure indicates that the phosphorylation of S44 leads to an increase in correlated motions between the various subdomains. Specifically, in the pS44 system, there is a strong correlation in intra-domain motions, whereas for WT, intra-domain motions exhibit a weak correlation. This suggests that phosphorylation promotes correlated motions within the domains. Regarding the overall correlated motions between the different subdomains, we observed that in both systems, the lyase and N-subdomain motions are not correlated, which is consistent with our previous study in the absence of DNA. In WT, the lyase-D subdomain motion is weakly correlated, but after phosphorylation, they move in opposite directions, which contradicts our previous observation. Additionally, in pS44, we observed more inter-domain correlated motions in different regions (highlighted by a blue dashed line square) compared to WT. The phosphorylation of S44 increased the correlated motions between DNA and different domains of the protein. As a result of S44 phosphorylation, both correlated and anti-correlated domain motions were modified. Notably, the cross-correlation map justified all the interactions explained in the previous section, such as the formation of salt bridges.

### 2.4. Principal Component Analysis

We performed principal component analysis by diagonalizing the covariance matrix of atomic fluctuations for both WT and pS44. The results are presented in terms of eigenvalues (3N = 3 × 354 = 1062, 326 $C_{\alpha }$, and 28 P atoms for DNA) and eigenvectors. The first 20 eigenvalues for both systems are shown in Figure S7 (in SI), plotted in descending order. The first few eigenvalues correspond to the largest fluctuations of the protein. The first 20 eigenvectors describe the collective motion of the DNA polymerase complex and account for approximately ∼91.5% and 82.6% of the overall motions in pS44 and WT, respectively. The first eigenvector covers around ∼28.5% and ∼66.9% of the total motions in pS44 and WT, respectively. For pS44, the first principal component is sufficient to describe the motion of the phosphorylated complex. We observe that the first few principal components that describe cooperative motion in pS44 are larger than those in WT. Furthermore, the magnitude of the first few eigenvalues in pS44 is much greater than in WT. The first two eigenvectors account for approximately ∼76% and ∼45% of the total motions in pS44 and WT, respectively. In contrast, in the absence of DNA, these values increase for both pS44 (∼79%) and WT (∼55%). The increase in overall motion that corresponds to the first two principal components in the absence of DNA suggests that DNA stabilizes the pol β complex. Overall, our results indicate that phosphorylation enhances correlated motion in the pol β complex, consistent with our cross-correlation results.

**Figure 8:**
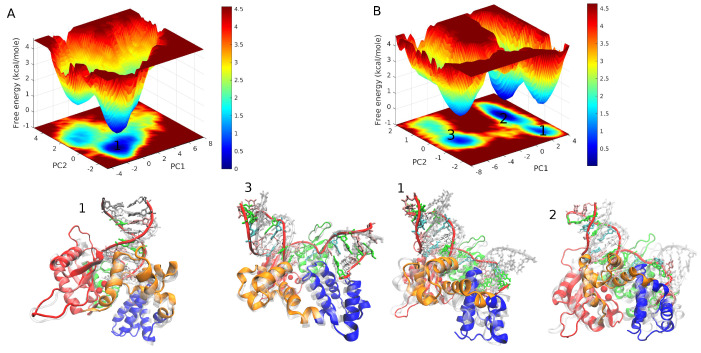
Free energy landscape of DNA polymerase β projected along the principal component 1 (PC1) and principal component 2 (PC2) (**A**) WT and (**B**) pS44. Representative structures corresponding to the minimum energy basins are also shown. For pS44, minimum energy basins are highlighted using the numbers.

We utilized PCA analysis to investigate the impact of phosphorylation on the FEL of pol β complex, which involves extracting the essential dynamics of the system from the MD trajectory, as described in the method section. The first two principal components were employed to generate FEL contour maps for both WT and pS44, as shown in Figure 8. The contour map for pS44 shows the system visiting multiple minimum free energy basins with large structural distributions, while the WT system has a single broad global minimum corresponding to the closed state of pol β complex. The minimum energy basins observed in pS44 are labeled as 1, 2, and 3. The structures in these basins are shown in snapshots and contrasted with the closed state of the enzyme. The snapshot in basin 1 depicts a pS44 system where the DNA binding domain is twisted due to the twist in the linker loop of the lyase domain. The snapshot in basin 2 corresponds to the twisting of the D-subdomain and opening of the N-subdomain. Basin 3 corresponds to the closed state. The results suggest that phosphorylation increases the flexibility and dynamics of the pol β complex.

**Figure 9:**
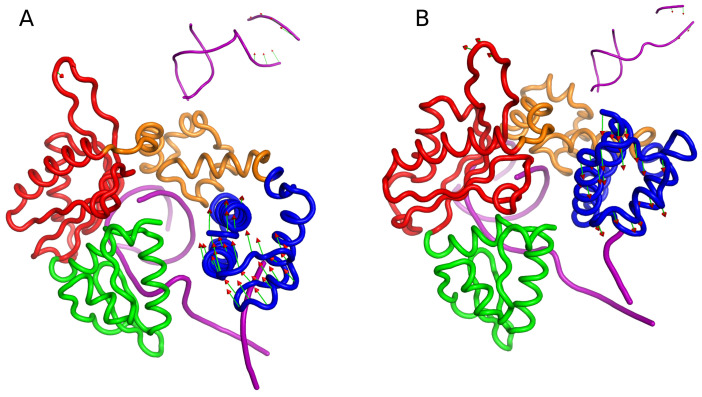
Procupine plots showing prominent motion for (**A**) pS44 (PC1), (**B**) WT (PC1). The inset of (**A**,**B**) show the prominent motion of DNA fragments. The arrow represents the eigenvectors showing the direction of prominent motions of pS44 and WT, respectively.

To illustrate how phosphorylation affects the direction and extent of different subdomain motions in the pol β complex, we visualized the eigenvectors corresponding to mode 1 using porcupine plots for both pS44 and WT. Porcupine plots were generated using Pymol, and the plots correspond to the largest collective motions of $C_{\alpha }$ atoms for the protein and P atoms for DNA, which had been mapped onto the average structure. A cutoff of 15 Å was chosen for the mode vectors to ensure clear visualization. As shown in Figure 9, phosphorylation changes the direction of motion of different domains of the pol β complex, which is consistent with the RMSF plot. The DNAs are shown in zoomed-in insets, and both figures are presented in the same orientation. The figure illustrates that for pS44, PC1 corresponds to the lyase domain moving closer to the C-subdomain, potentially hindering the alignment of the dNTP with the insertion gap, while for WT, it corresponds to the opening of the lyase domain. The movies of both systems for PC1 are available in the supplemental information. Moreover, Supplementary Figure S8 (in Supplementary Materials) depicts the residue-wise contribution of PC1 for both pS44 and WT. The figure illustrates that the presence of an additional phosphate group in the phosphorylated enzyme leads to a more compact conformation of the complex, driven by the DNA.

### 2.5. Phosphorylation of S44 Changes the Influence/Sensitivity Profile of DNA Polymerase β Complex

**Figure 10:**
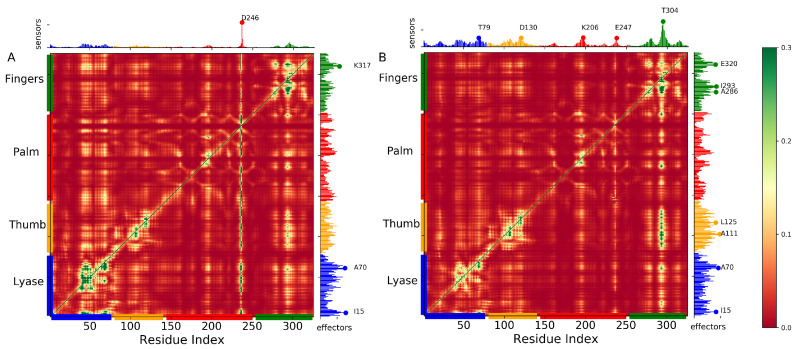
Perturbation response analysis identifies the highly influential and sensitive residues that likely propagate allosteric signals in WT and pS44. PRS maps ((**A**) WT and (**B**) pS44) indicate the strongest perturbation response sites as shown by the color map (see the scale on right). The peaks in the curves along the axes indicate the effectors (left ordinate) and sensors (upper abscissa). DNA polymerase β subdomains are color-coded along the lower abscissa highlighting lyase (blue), thumb (orange), palm (red), and finger subdomains (green), as mentioned in Figure 1.

To investigate the impact of phosphorylation on the long-range transmission signals, we utilized the perturbation response scanning (PRS) method on both pS44 and WT systems. The PRS map was constructed using the ANM model’s first 20 nonzero frequency modes, which reveal cooperative changes in the structure close to the native state. The PRS method (explained in the method section) probes the response of each residue to perturbation in every other residue. Figure 10 displays the PRS maps of both systems, which were drawn using the MD trajectory. Each element in the PRS matrix represents the response of residue *j* to a perturbation on residue *i*.

The rows of the PRS map provide information about the influence or effectiveness of a particular residue in transmitting signals when subjected to unit perturbation, while the columns provide information about the sensitivity of a given residue to those signals. The effector residues (peaks in the right ordinate bar plot) and sensor residues (peaks in the upper abscissa bar plot) are colored by domain/subdomain identity in the panel.

The results show that the background response of the DNA complex remains relatively unchanged due to phosphorylation (S44), but specific regions show significant changes. In the case of WT, there is a strong enhancement in sensitivity for residue D246 (Catalytic domain), but the signals in the other subdomains are weak. However, due to phosphorylation, the sensitivity at D246 is suppressed, and a new signal emerges, as shown in Figure 10. The most affected sensors due to phosphorylation clustered in the catalytic domain and N-subdomain, while the effectors clustered in the lyase domain and N-subdomain. Additionally, due to phosphorylation, a few new signals (A111, and D130) emerge in the DNA binding domain. The structural analysis of the phosphorylation’s effect on the lyase domain may be further supported by this observation, as it suggests that the N- and C-subdomains are indeed disrupted.

### 2.6. Structural Network Analysis

In order to understand the functional movements and information exchange mechanism, we utilized a protein network method known as residue network analysis (RNA) on our MD trajectory. The RNA methodology involves a weighted network where the nodes represent the $C_{\alpha }$ atoms of amino acids in the protein and the phosphate atom in DNA, which are connected by edges whose weights are based on the correlation between residue pairs. Since the dynamic cross-correlation method utilizing the Pearson correlation coefficient may miss some correlations, we used the linear mutual information (LMI) approach [32,33] to compute the RNA. The LMI-based residue network is utilized for conducting centrality analysis. Centrality analysis has been extensively employed to determine the functionally significant residues in communication pathways, metabolic networks, disease networks, and allosteric communications [34,35,36,37]. The three most commonly utilized centrality measures are degree (deg), closeness (CL), and betweenness (BC). We calculated the centralities for both the WT and pS44 complexes to investigate the impact of phosphorylation on the communication pathways connecting the regions that are crucially involved in the functional movements of the system.

**Figure 11:**
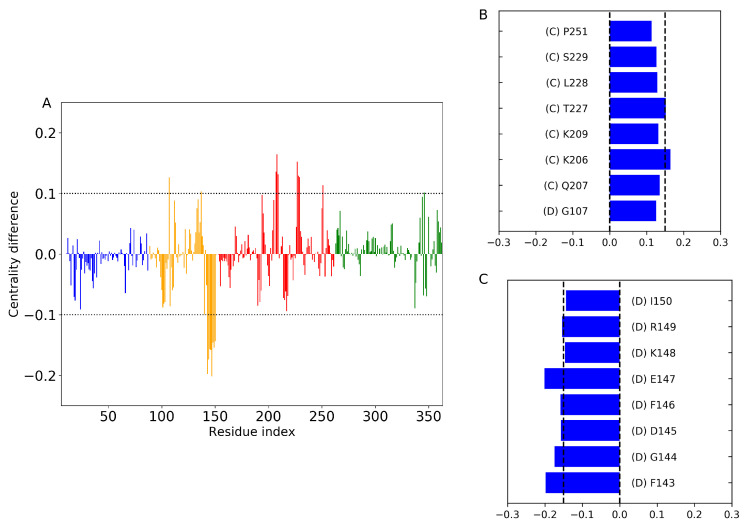
(**A**) The values of the difference in betweenness centrality with respect to WT plotted as a function of residue index. The horizontal dotted lines correspond to the value ±0.1. The data are colored according to domain/subdomain color as shown in Figure 1. Residues with large changes in normalized BC value due to the phosphorylation (**B**) largest positive change and (**C**) negative change. The DNA pol β subdomains corresponding to those residues are also mentioned next to the residue number.

Previous studies [38] have demonstrated that a residue with a high BC value is closely related to the functional residue that conveys the allosteric signal, highlighting the importance of BC over the other two centrality measures [39]. In our study, we employed the difference in BC as a metric to identify the key residues that experience changes in relative importance along the information pathway. The normalized BC difference was calculated by comparing the phosphorylated system with the wild type, i.e., $BC^{phos}-BC^{WT}$, for each residue in the protein and phosphate atom of DNA using our all-atom trajectory. Figure 11A illustrates the difference in BC values per residue for the phosphorylated system in comparison to the wild type. The results indicate that some residues lose and others gain importance along the information pathway.

The C-subdomain of the phosphorylated system shows significant changes (>0.1) in BC values for most residues, except for G107 in the D-subdomain, as shown in Figure 11B,C. This indicates that the C- and D-subdomains are critical for information flow and that mutations in this region can disrupt the allosteric network and impact the function of the DNA pol β complex.

The most noteworthy finding is the substantial change in BC observed in residues K206 and T227. Additionally, a significant decrease in BC is observed in residues F143 to I150, which correspond to the loop region connecting the DNA binding subdomain (D) to the catalytic subdomain (C). This and the Lyase domain moving closer to C-subdomain may support the notion that phosphorylation causes the lyase domain to disrupt the proper alignment of the catalytic region (K206 to K209) highlighted by the BC calculation aligns with the sensitivity profile of pS44 observed in the PRS calculation, further supporting our results.

## 3. Materials and Methods

In this study, the closed form of DNA pol β ternary complex was taken from crystallographic structure (PDB ID: 2FMS [30]). The system is composed of DNA, proteins, and ions. To explain the underlying mechanism of observed conformational changes, the following systems were investigated: (1) unphosphorylated DNA pol β complex designated as WT, and (2) phosphorylated DNA pol β complex, where serine-44 (S44) was replaced by a phosphorylated residue from (SP2 patch), designated as pS44. The modeled structure included all the ions which were present in the crystal structure. All the Mg ions were placed at the same positions as resolved in the crystal structure whereas Na ions were placed randomly. In order to understand the mechanism of phosphorylation-induced conformational changes in DNA pol β, we have simulated the above systems for 1 μs. To compare how the Mg ions stabilize the WT system, we have also simulated the WT system in the absence of Mg ions.

### 3.1. Simulation Protocol

The GROMACS 2020 software package [40] was used to carry out all-atom molecular dynamics (MD) simulations under constant temperature and pressure (NPT) conditions. The Charmm36 force field [41] parameters were used both for protein and DNA, in addition to the TIP3P water model [42]. The system was solvated in a cubic box with a distance of 10 Å from the box walls. Ions were added to neutralize the systems. The integration of the equation of motion was performed using a 2.0 fs time step. To maintain a constant temperature of 300 K, the Berendsen thermostat was employed. The pressure was iso-tropically maintained at 1 bar using the Parrinello–Rahman barostat [43]. Periodic boundary conditions were applied in all three directions. A cutoff radius of 10 Å was used for neighbor search. The neighbor list for non-bonded pairs was updated every 40 steps. The short-range interactions are truncated after 10 Å with dispersion correction. Long-range electrostatic interactions were computed using the particle mesh Ewald summation method [44] with a grid spacing 0.16 nm and interpolation of order 4. SETTLE [45] and LINCS algorithms [46] were used to constrain the covalent bonds of the system to their equilibrium geometries. To eliminate unfavorable contacts that may arise from the random distribution of water and ions, each system underwent a gradual energy minimization using the steepest descent minimization method for 10,000 steps. After the energy minimization, the systems were equilibrated in the NVT ensemble at a temperature of 300 K with positional restraints for 10 ns to ensure stability. Subsequently, the systems were equilibrated in the NPT ensemble at a pressure of 1 bar and a constant temperature of 300 K for a total of 10 ns. We conducted a 1000 ns (1 μs) production run for each system under unrestrained NPT conditions at 300 K and 1 bar, with coordinates recorded every 2 ps for subsequent analysis. A schematic diagram of the simulated system is depicted in Figure 1.

### 3.2. Structural Analysis and Visualization

The built-in GROMACS functions [40] such as gmx rms, gmx gyrate, gmx rmsf, and gmx hbond were used for the structural analysis of the trajectory produced for each DNA pol β complex system after MD simulation. We used these functions to compute the root mean square deviation (RMSD), the radius of gyration ($R_{g}$), root mean square fluctuations (RMSF), and the number of hydrogen bonds (H-bonds). For the H-bonds, we used the following criteria: the distance between the donor and acceptor is *d* ≤ 3.5 Å, and the angle between the donor and acceptor is >$30^{0}$.

The results were plotted using the matplotlib v3.3.2 [47]. The structural images were produced using visual molecular dynamics (VMD) [48] and PyMOL [49].

### 3.3. Dynamic Cross-Correlation

The dynamics cross-correlation coefficient (DCCM) [50] between the *i*th and *j*th atoms is defined by the following equation,

(1) $$C\left(i,j\right)=\frac{<\left(r_{i}\left(t\right)-<r_{i}\left(t\right)>\right)\cdot (r_{j}\left(t\right)-<r_{j}\left(t\right)>){>}_{t}}{{\left(<r_{i}^{2}\left(t\right)>-<r_{i}\left(t\right){>}^{2}\right)}_{t}^{1/2}{\left(<r_{j}^{2}\left(t\right)>-<r_{j}\left(t\right){>}^{2}\right)}_{t}^{1/2}}$$

where $r_{i}\left(t\right)$ and $r_{j}\left(t\right)$ denotes the vector of the *i*th and *j*th $C_{\alpha }$ atom position as a function of time *t*. The term “$r_{i}\left(t\right)-<r_{i}\left(t\right)>$” represents the deviation or fluctuation of the position of the *i*th atom from its average position, denoted by “$<r_{i}\left(t\right)>$”. Similarly, the term “$r_{j}\left(t\right)-<r_{j}\left(t\right)>$” corresponds to the deviation of the position of the *j*th atom from its average position.

The cross-correlation metric elements do not convey information regarding the magnitude of $C_{\alpha }$ atom motions. The sign of C(i,j) denotes whether the motions are correlated or anti-correlated, where positive values indicate correlated motions and negative values indicate anti-correlated motions. A C(i,j) value of 1 indicates perfectly correlated motion, whereas a value of −1 indicates perfectly anti-correlated motions. Perfect correlation occurs when two atoms move in the same direction with the same phase and period, while a value of 0 occurs when two atoms move with the same period and phase, but their displacements are oriented at a 90${}^{\circ }$ angle.

### 3.4. Principal Component Analysis

Principal component analysis (PCA) is a mathematical technique used for the dimensionality reduction of large datasets. It transforms a large number of variables into a smaller set of variables while retaining most of the original information. PCA or essential dynamics has been applied in proteins to extract essential information about the nature and directions of protein motion from a set of conformations generated by MD simulations [50].

PCA is a mathematical technique that involves diagonalizing the covariance matrix whose elements are denoted by $\Gamma_{ij}$. The covariance matrix is constructed using the Cartesian coordinates of the structures generated from the MD simulations:

(2) $$\Gamma_{ij}=<\left(r_{i}-<r_{i}>\right)\left(r_{j}-<r_{j}>\right)>,$$

where $r_{i}$ and $r_{j}$ represent the X-, Y-, and Z-coordinates associated with all possible pairs *i* and *j*, respectively. Here in this work, the PCA was performed on the $C_{\alpha }$ atoms of protein and phosphate atoms of DNA. The covariance matrix [Γ] obtained from the atomic fluctuations of protein is diagonalized as:

(3) $$\left[\Gamma \right]=U\Lambda U^{T}$$

where Λ represents the diagonal matrix of eigenvalues and *U* represents the matrix of eigenvectors. The eigenvectors and eigenvalues obtained from PCA represent the principal components that can be used to describe the motions of the system. The eigenvalues indicate the magnitude of the motion, while the eigenvectors indicate the direction of the motion. In this study, the first 20 eigenvectors were analyzed, and the two eigenvectors designated as PC1 and PC2 have a cosine content less than or equal to 0.1. These were used to generate the FEL using the gmx sham function in GROMACS, which computes the minimum free energy configuration from PC1 and PC2. The FEL maps were generated using MATLAB [51].

### 3.5. Perturbation Response Scanning

The perturbation response scanning (PRS) technique [52] was employed to identify the most important and sensitive residues of DNA polymerase β using the anisotropic network model (ANM) implemented in PRoDy [53]. ANM [54] represents the protein as a network of nodes corresponding to the positions of $C_{\alpha }$ atoms of each residue, and the inter-residue interactions are modeled using harmonic springs within a threshold distance of 12 Å. PRS is based on linear response theory, and it identifies the effects of perturbing each residue by measuring the changes in the protein’s dynamics. The Hessian matrix (*H*) obtained by ANM potential is used to define the collective dynamics of the network. In PRS, each residue in the network is sequentially subjected to a force of a fixed magnitude, and the resulting response of the overall network is observed. The ANM is used to calculate the displacement of each residue in response to the applied force using Hook’s law (*F* = HΔR or ΔR =$\;H^{-1}F$), which relates force to displacement. The displacement of all residues, $\Delta R^{\left(i\right)}$, is computed in response to the applied force on each residue, *i* = 1 to *N*. Allosteric sensors and effectors are identified using the method proposed by General et al. [55]. The PRS analysis is based on the first 20 slowest non-zero eigenvalues of the ANM modes, which describe the most energetically favorable collective changes in the protein structure.

### 3.6. Structural Network Model

The betweenness centrality (BC) is a graph theoretical method used to quantify the amount of information flowing through the nodes and edges of a network. In our residue interaction network, each node represents the $C_{\alpha }$ atom of amino acid and phosphate atoms for nucleotides. The edges connecting the nodes represent the correlation between them. If a node *i* acts as a bridge between two other nodes along the shortest path joining them, then the BC of node *i* is given by the equation:

(4) $$BC\left(i\right)=\sum_{ab}\frac{n_{ab}^{i}}{g_{ab}}$$

Here, $g_{ab}$ represents the total number of shortest paths joining nodes *a* to *b*, out of which $n_{ab}^{i}$ paths pass through node *i* [56]. By analyzing the BC values, we can identify the residues that are involved in the information-propagating pathway. In this study, we use the difference in BC values as a metric to identify the key residues that gain or lose relative importance along the information pathway due to phosphorylation.

## 4. Conclusions

Our MD simulations have revealed that the phosphorylation of serine 44 in DNA polymerase has led to significant structural changes that could potentially inhibit the enzyme’s activity. These findings are in line with our previous research on S44 phosphorylation [23] and could explain the enzyme deactivation of polymerase catalytic function observed by Touki et al. [21]. The impact of S44 phosphorylation on the apoenzyme and its complex with gapped DNA shares several similarities, primarily driven by electrostatic effects. These include the disturbance of the locking hydrogen bond between S44 and E335 and the formation of new salt bridges. The impact of S44 phosphorylation on the conformation of Pol β differs considerably depending on whether DNA is present or not. In the absence of DNA, the enzyme tends to adopt a more open conformation, with the lyase domain swinging away from the N-subdomain and resulting in a more expanded structure. On the other hand, in the presence of DNA, the phosphorylated Pol β adopts a more compact conformation, with the lyase domain collapsing towards the C-subdomain. The analysis techniques employed in this study consistently indicate that the pS44 complex has a more compact conformation. The Rg and RMSD distributions as well as the FEL all demonstrate a shift from the closed structure of the WT to a structure in which the lyase domain is twisted towards the catalytic subdomain, resulting in a higher RMSD and lower Rg values compared to the closed state. This reorientation of the lyase domain is responsible for the observed compactness in the pS44 system. Furthermore, the increase in the number of hydrogen bond formations within the different subdomains of the pS44 system indicates an increased intradomain rigidity. This observation is further supported by cross-correlation analysis. Moreover, the formation of a new salt bridge between S44 and R149 provides additional evidence for the tendency of the lyase domain to move closer to the C-subdomain. Principal component analysis (PCA) confirms that the dominant components in the pS44 system involve inward movement of the lyase domain as well as increased inter-domain flexibility, as reflected in the greater number of basins observed in the PC1–PC2 free energy landscape. The impact of these structural changes is also evident in the PRS and network analyses.

The extensive conformational changes induced by S44 phosphorylation are expected to have significant implications for the enzyme’s function. The interference between the lyase domain and the C-subdomain may lead to a disruption in the proper alignment of the DNA fragment with the nascent incoming dNTP, as this alignment occurs within the catalytic subdomain [18]. Adding a negative charge to S44 is postulated to interfere with the positive charge on R149 resulting in a catalytic failure of the enzyme. Furthermore, the negative charge on the phosphorylated S44 may interfere with repositioning the N-subdomain that occurs when the enzyme binds to nucleotides. Furthermore, the lyase domain plays a crucial role in searching for damage sites on the DNA through a hopping mechanism [57]. The collapse and re-orientation of the lyase domain upon DNA binding to pS44 pol β may hinder its scanning function for DNA damage as it may disable enzymes for hopping along the DNA strand to search for damaged sites, thereby interfering with BER. In summary, this study offers new insights into the mechanism of enzyme inactivation resulting from S44 phosphorylation.

## Data Availability

The data are available upon reasonable request from the authors.

## Supplementary Materials

The supporting information can be downloaded at: https://www.mdpi.com/article/10.3390/ijms24108988/s1.
