# Supplementary material for: Structural Insights into Phosphorylation-Mediated Polymerase Function Loss for DNA Polymerase β Bound to Gapped DNA
Source: Int J Mol Sci. 2023 May 19;24(10):8988. doi: 10.3390/ijms24108988 (PMC10219072; doi:10.3390/ijms24108988)

Article

Supplementary Information for Structural Insights into Phosphorylation-Mediated Activity Loss in DNA Polymerase β Binding to Gapped DNA

Amit Srivastava [1], Haitham Idriss [2,3,4] and Dirar Homouz [1,*]

[1] Department of Physics, Khalifa University of Science and Technology, 127788 Abu Dhabi, UAE
[2] School of Public Health, Imperial College of Science, Technology and Medicine, London SW7 2AZ, UK; haitham.idriss19@imperial.ac.uk
[3] Palestinian Neuroscience Initiative, Al-Quds University, Jerusalem 51000, Palestine
[4] Faculty of Health Sciences, Global University, Beirut 15-5085, Lebanon
[*] Correspondence: dirar.homouz@ku.ac.ae; Tel.:+971 2 312 4154

Citation: Srivastava, A.; Idriss, H.; Homouz, D. Structural and Dynamical effect. *Preprints* **2023**, *24*, 8988. https://doi.org/10.3390/ijms24108988

Publisher's Note: MDPI stays neutral with regard to jurisdictional claims in published maps and institutional affiliations.

Copyright: © 2023 by the authors. Submitted to *Preprints* for possible open access publication under the terms and conditions of the Creative Commons Attri- bution (CC BY) license (https://creativecommons.org/licenses/by/4.0/).

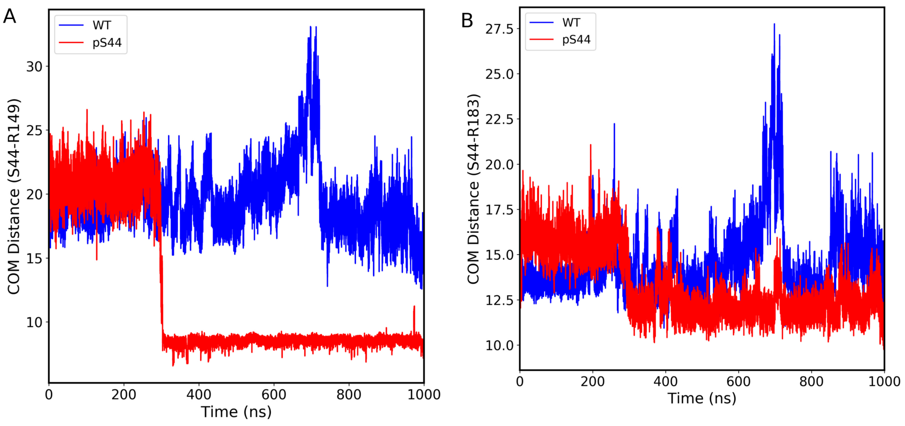

Figure S1. Distance *vs* simulation time (**A**) Center of mass distance between S44-R149. (**B**) Center of mass distance between S44-R183.

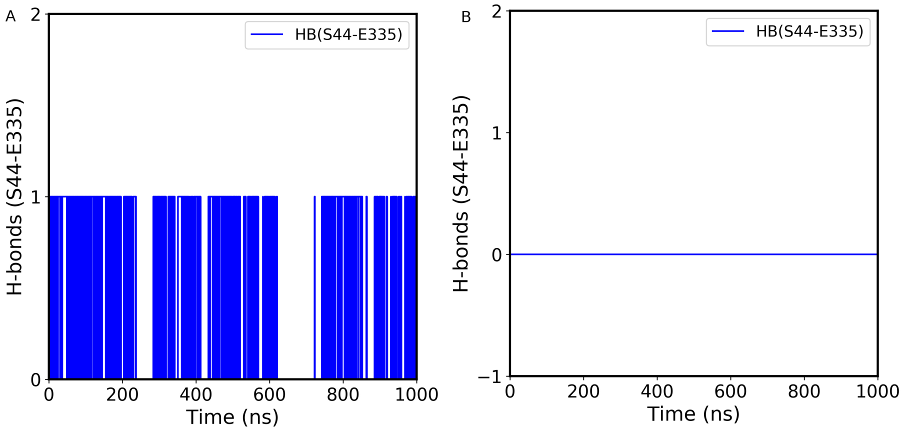

Figure S2. Time evolution of hydrogen bond between S44-E335 (**A**) WT, (**B**) pS44

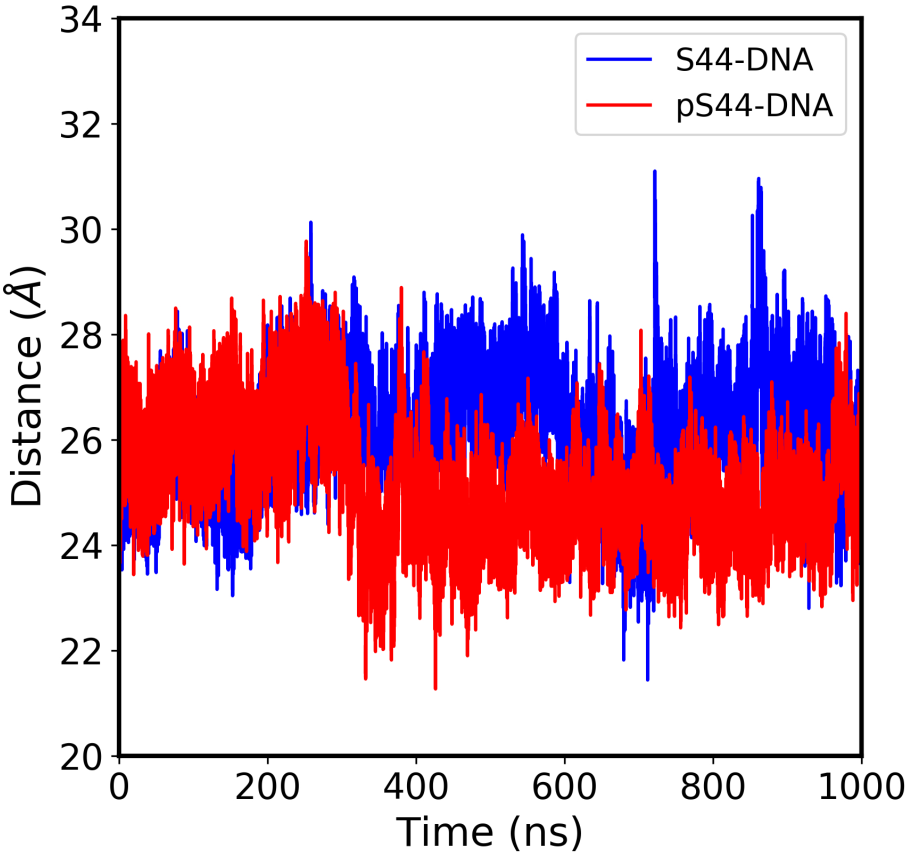

Figure S3. Distance between S44 and DNA fragment *vs* simulation time. The blue solid line shows the distance between S44 and DNA, whereas, the red line shows the distance between phosphorylated S44 residue and DNA.

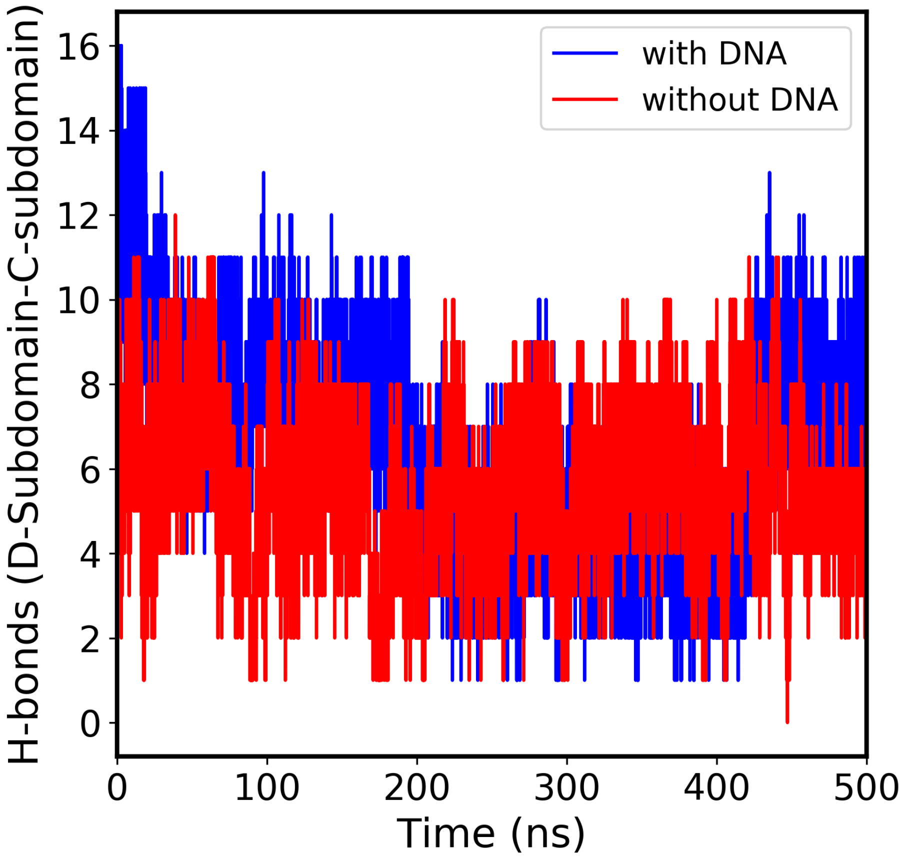

Figure S4. Time evolution of hydrogen bond between DNA binding -Catalytic subdomains in presence and absence of DNA.

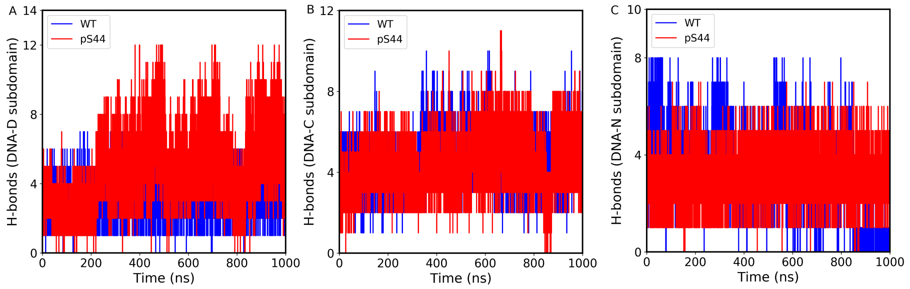

Figure S5. Time evolution of Hydrogen bonds (**A**) DNA and DNA binding subdomain, (**B**) DNA and Catalytic subdomain, and (**C**) DNA and N-base pairing subdomain. The blue solid line shows the hydrogen bond for WT, whereas, the red line shows the hydrogen bond for pS44.

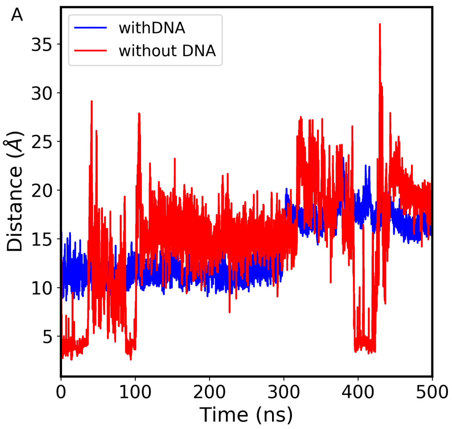

Figure S6. Distance *vs* time (**A**) pS44-K280; with and without DNA fragment, (**B**) pS44-R299; with and without DNA fragment.

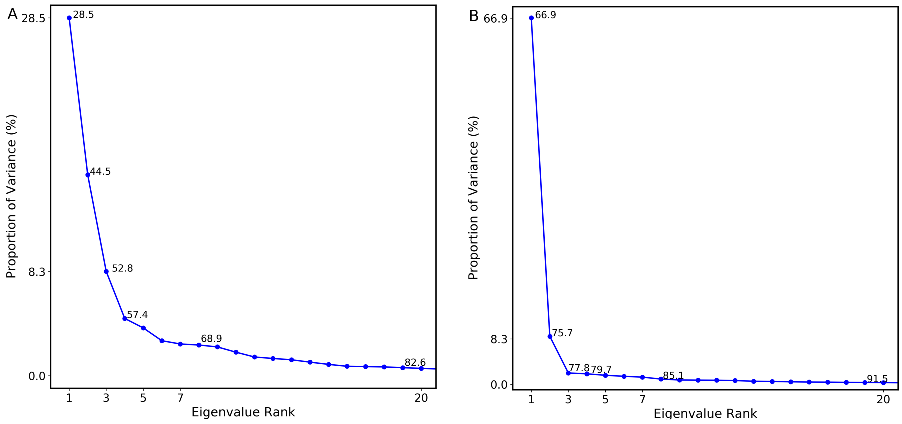

Figure S7. Scree plot for principal component analysis on the MD data of DNA pol β complex (**A**) WT, and (**B**) pS44.

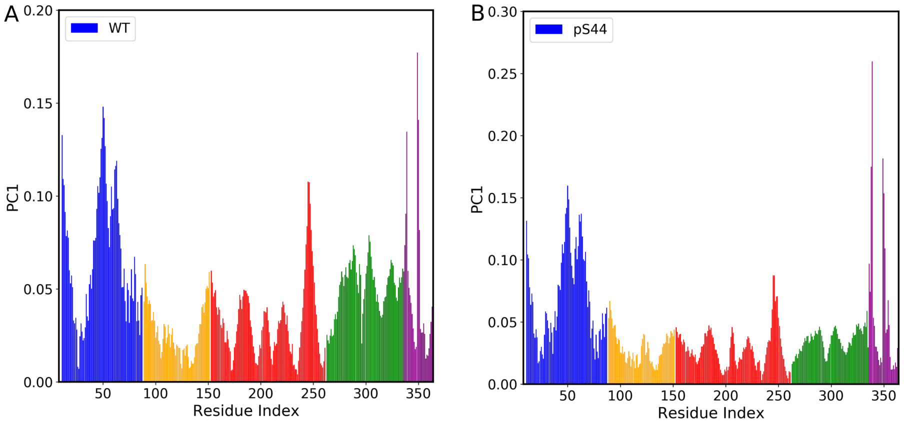

Figure S8. DNA polymerase β complex each residue and nucleotide contribution to principal component 1 (PC1) of (**A**) WT, and (**B**) pS44. Different colors in the figure represent the different domains and subdomains of DNA pol β complex as shown in figure 1 (in the main text).

Table S1. The donor-acceptor distance between S44 and E335 for three systems.

System	Average dist (Å)	St. Dev. (Å)
WT	6.18	2.10
phosphorylated S44	11.3	2.31
pS44	10.53	1.72

frame **Movie of Principal Component 1 for WT.** 1

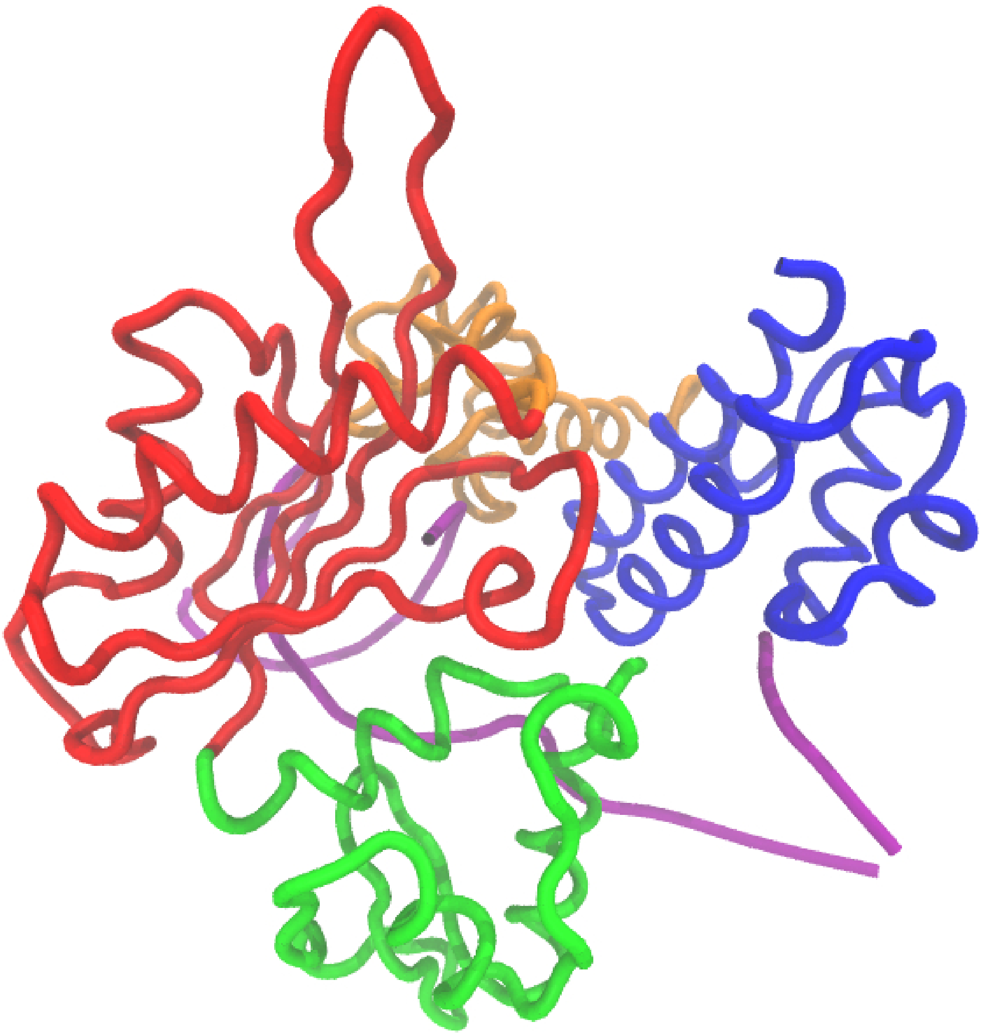

frame **Movie of Principal Component 1 for phosphorylated S44.**

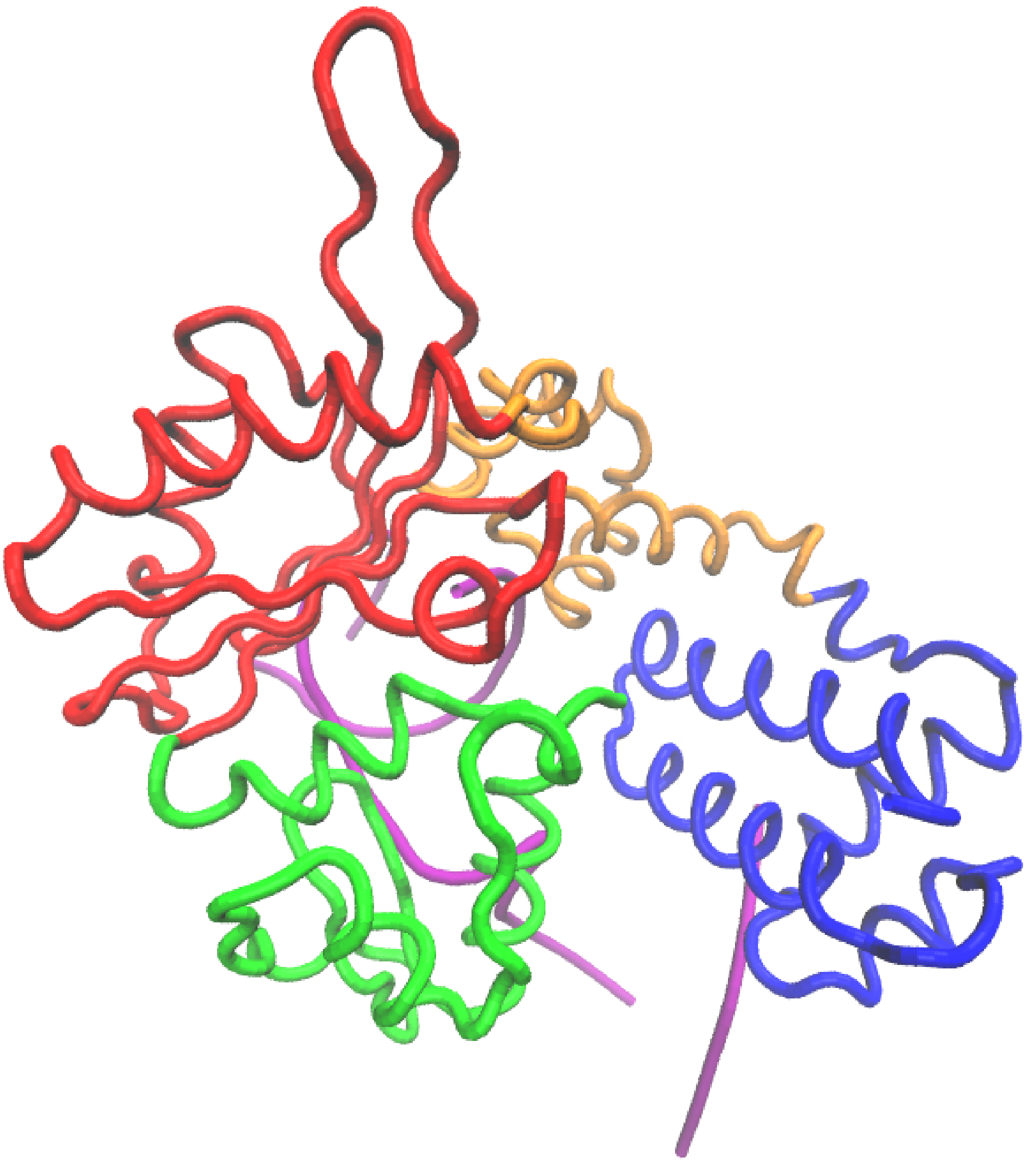

Supplement: Supplementary file 1 [file ijms-24-08988-s001.zip › ijms-2378434-supplementary/ijms-2378434-supplementary.pdf]
